# Ferritin family proteins and their use in bionanotechnology

**DOI:** 10.1016/j.nbt.2014.12.006

**Published:** 2015-12-25

**Authors:** Didi He, Jon Marles-Wright

**Affiliations:** Institute of Structural and Molecular Biology, School of Biological Sciences, University of Edinburgh, Mayfield Road, Edinburgh EH9 3JR, United Kingdom

## Abstract

Ferritin family proteins are found in all kingdoms of life and act to store iron within a protein cage and to protect the cell from oxidative damage caused by the Fenton reaction. The structural and biochemical features of the ferritins have been widely exploited in bionanotechnology applications: from the production of metal nanoparticles; as templates for semi-conductor production; and as scaffolds for vaccine design and drug delivery. In this review we first discuss the structural properties of the main ferritin family proteins, and describe how their organisation specifies their functions. Second, we describe materials science applications of ferritins that rely on their ability to sequester metal within their cavities. Finally, we explore the use of ferritin as a container for drug delivery and as a scaffold for the production of vaccines.

## Highlights

• We discuss bionanotechnology applications of ferritin family proteins.
• Ferritin family proteins are able to mineralise a range of metal ions.
• The ferritin and DPS cages can be used in semi-conductor patterning.
• We explore a commercial application of ferritin as a phosphate removal system for water purification.
• We examine how the superparamagnetic properties of iron-loaded ferritin can be used in medical imaging.

## Introduction

Nanotechnology concerns itself with materials in the 1–100 nm size range; at this scale materials exhibit remarkably different properties to bulk materials. Graphite is the most common naturally occurring allotrope of carbon and has been used by humans for over 500 years, yet in single layers it is the 21st century wonder-material graphene [1]. In bionanotechology, biological systems as varied as viruses, protein complexes, lipid vesicles and artificial cells, are being developed for applications in civil engineering, medicine and materials science [2,3]. The capsids of viruses and other protein complexes, such as ferritins and heat shock proteins, with defined interior cavities are particularly attractive targets for bionanotechnology: they are readily produced in large quantities; have well-characterised atomic structures; are usually monodisperse in solution; and are amenable to chemical and biological modification [4,5].

The ferritin family proteins are ubiquitous in nature and have been the subject of much research focused on their applications in bionanotechnology. Almost 3000 published patents mention ferritin and nanotechnology, 100 of which specifically mention bionanotechnology. The primary role of ferritin is to protect cells from the damage caused by the Fenton reaction; where, in oxidising conditions, free Fe(II) produces harmful reactive oxygen species that can damage the cellular machinery [6]. Ferritins are also able to store a significant quantity of iron within a hollow core, and act as storage systems for iron within cells [7].

The active site of ferritin family proteins, named the ferroxidase centre (FOC), is able to safely oxidise iron (II) in the presence of oxygen, or peroxide, to produce ferrihydride minerals that are stored within the core of the ferritin nanocage [8]. The FOC active site has conserved glutamic acid, aspartic acid and histidine residues that coordinate iron and facilitate its controlled oxidation [9]. Because ferritin family proteins are able to mineralise and store metal ions, they have been the focus of much research for the production of metal nanoparticles [10,11], and as templates for semi-conductor production [12].

The ferritin cage itself is highly symmetrical, and is made up of 24 subunits arranged in an octahedral (432) symmetry. The ferritin cage displays remarkable thermal and chemical stability and it is particularly amenable to reconstitution through controlled (dis-)assembly [13]; it is also possible to modify the surface of the ferritin cage through the addition of peptide and protein tags [14]. These characteristics have made ferritins attractive vectors for the delivery of drug molecules [15] and as scaffolds for vaccine design [16]. In Table 1 we summarise the bionanotechnology applications of ferritin covered in this review, and highlight the source of the ferritin and features of the protein that are exploited in each application. In this review we will explore how the structural and functional features of ferritin nanocages have been applied in materials science to make use of their natural ability to sequester metal; and in the biomedical field, where modified ferritin proteins are used as drug delivery vehicles, and as scaffolds for vaccine development. Each application will be put into context and considered in contrast to alternative methods to reach the same ends. Finally, we will consider the scope for commercialising and exploiting the inventions highlighted in this review.

## Ferritin family proteins

**Figure 1:**
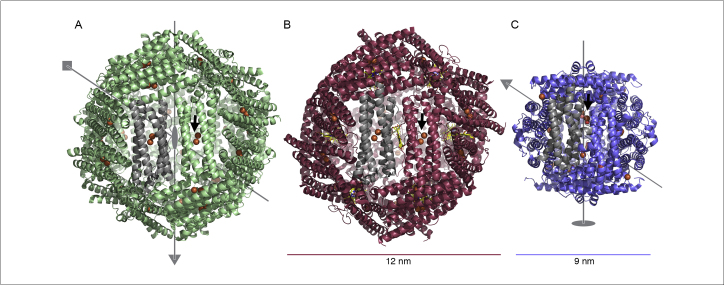
Structure of ferritin family protein nanocages. The quaternary structures of the three ferritin family protein nanocages are shown as cartoon representations, a single monomer is coloured grey and bound iron ions in ferroxidase active sites are shown as orange spheres. **(a)** Structure of ferritin from *Pseudonitzchia multiseries* (PDB ID: 4IWJ) [17]. Grey lines and polygons depict the relationship between the symmetry axes of the ferritin cage: 2-fold, ellipse; 3-fold, triangle; 4-fold, square. **(b)** Structure of bacterioferritin from *Desulfovibrio desulfuricans* (PDB ID: 1NF4) [71]. The bound haem-b co-factors are shown as yellow sticks between the protein monomers; due to the co-ordination of this group the 2-fold axes between monomers are not true symmetry axes. Bacterioferritin is shown in the same orientation as ferritin. **(c)** Structure of DPS from *Microbacterium arborescens* (PDB ID: 2YJK) [22]. DPS is a dodecamer and has 2- and 3-fold symmetry axes; the relationship between these is illustrated with grey lines and a triangle and ellipse.

Before summarising the applications of the ferritin family proteins we must introduce the various family members and highlight their structural and functional differences. The classic ferritin (**Ftn**), found in eukaryotes and some bacteria, is a four-helix bundle protein of around 200 amino acids that forms a cage of 24 subunits arranged in octahedral 432 symmetry [17], with an outer diameter of roughly 12 nm and an inner diameter of 8 nm (Fig. 1a). Mammalian ferritins are heteropolymers consisting of an H-chain subunit (182 amino acids, containing the ferroxidase centre) and an L-chain subunit (174 amino acids, facilitating the mineralisation of Fe^3+^) [18]. Other Ftn variants exist including the amphibian M-chain ferritin, which has the FOC active site, but is shorter than H-chain Ftn [9]

Along with the classic ferritin, some bacteria and archaea possess bacterioferritin (**Bfr**), which differs from Ftn by the incorporation of twelve b-type haem groups between subunits in a twofold symmetric binding site [19] (Fig. 1a). The role of the haem group is not completely clear, although recent work has shown that it may act to allow electron transport from Bfr-associated ferredoxins to the Bfr core to mobilise stored iron [20]. The Bfr-associated ferredoxins possess a [2Fe-2S] cluster that is positioned above the haem group when the protein binds to Bfr, this molecular-wire allows electrons to be passed from cofactors, such as NAD(P)H, to the iron core to reduce the mineralised iron [20].

The mini-ferritin, DNA-binding Protein from Starved cells (**DPS**), was initially discovered in *Escherichia coli* cells during the stationary phase and is found in many bacteria and some archaea [21,22], and its primary role is to protect the bacterial chromosome from oxidative damage [25]. In contrast to the 24-meric assembly of Ftn and Bfr it has a distinct dodecameric assembly, with 9 nm outer and 5 nm inner diameters (Fig. 1c). Like Ftn and Bfr, DPS has a ferroxidase site and can store iron within its core, albeit in smaller quantities due to the smaller inner diameter of the dodecameric complex [23,24]. DPS expression is induced during stationary phase and it binds to the bacterial chromosome in a sequence-independent manner through interactions mediated by positively charged N-terminal tails. Furthermore, the complex self-associates to induce chromosome condensation [26]. Knockout of DPS genes in pathogens, such as *Bacillus anthracis*, reduces tolerance to oxidative stress and thus their virulence [27].

## Production of ferritin for bionanotechnology applications

The various nanotechnology applications of ferritin covered in this review use ferritin from a number of sources and in most cases only relatively small quantities of protein are used in these proof of concept projects. Very few industrial-scale processes use ferritin-based technologies, with the use of *Pyrococcus furiosus* ferritin in water treatment the most notable [28]. Ferritin family proteins are found in all kingdoms of life and as such are relatively easy to isolate and produce [29]. Horse spleen ferritin is available for roughly £200 a gram from major chemical suppliers, while human ferritin variants are more expensive at around £50 per microgram (prices taken from Sigma–Aldrich website, November 2014). Recombinantly produced bacterial and archaeal ferritin nanocages are readily produced at the milligram to gram scale in the laboratory (references in Table 1). It is possible to produce mammalian ferritin variants by recombinant methods, although the subunit composition varies from the native protein as the latter contains L- and H-chain variants in variable ratios, while the former are usually produced as just the active site containing H-chain variant [30].

Purification of recombinant ferritin and DPS nanocages is facile because these proteins are generally thermostable. Established protocols separate recombinantly produced ferritin by heating clarified *E. coli* cell lysate to around 70°C to denature the native *E. coli* proteins and leave only the thermostable ferritin in solution (references in Table 1). The denatured proteins are removed by centrifugation and the ferritin remains in the supernatant. Further purification steps, such as chromatographic separations and differential centrifugation, may be performed to produce material with very high purity. Both native and recombinant ferritin often contains a significant amount of iron within its mineral core, and depending on the final application this may not be desired. The iron core can be removed by reducing the iron with suitable reducing and chelating agents, such as sodium dithionite and EDTA, or BIPY [31].

### Ferritin as a biomineralisation scaffold

The ferritin family proteins are probably the best-studied biomineralisation scaffolds and are able to accommodate up to 4500 iron atoms in a ferrihydride form within the central cavity [32]. In the catalytic cycle of ferritin family proteins iron(II) is oxidised within the ferroxidase centre; the resulting iron (III) ions transfer to the central cavity and mineralise as Iron (III) hydrides where they form microcrystals [29,33]. Negatively charged pores in the ferritin shell, formed between subunits, allow the entry and exit of cations during mineralisation and demineralisation [34]. The selectivity of these channels is relatively broad and it is possible *in vitro* to induce the mineralisation of a variety of metal ions, with a strong preference for divalent cations [23], although ferritin will tolerate some mono- and trivalent metal ions [35]. In practice, the ferritin cage has been employed as a template to mineralise a range of different non-physiological metals and metal complexes through either self-assembly around solutions of metal ions, chemically mediated redox reactions, or photochemistry [5,36–38]. The conditions required for the production of various metal nanoparticle cores is summarised in Yoshimura's excellent review [10].

Early studies on the plasticity of ferritin mineralisation demonstrated the production of iron sulphide, Mn(III) oxide and magnetite (Fe_3_O_4_) cores [11]. The latter, ‘magnetoferritin’, has potential uses as a magnetic contrast agent for cell imaging and for the magnetic separation of labelled cells and particles [39]. Recent interest in the use of magnetoferritin has focused on using it as a label to target and visualise tumour cells [40]. The negatively charged interior and exterior surface of ferritin readily coordinates metal ions, although the case of gold ions the efficiency of mineralisation is reduced by these surface interactions. To get around this limitation reconstitution and washing of the ferritin cage has been used to optimise the production of gold nanoparticles [31]. Surface modification by mutagenesis has allowed the production of noble metal nanoparticles within the ferritin interior [32]. Co-crystallisation of metals and semi-conducting compounds has also been explored [12].

The range of possible metal nanoparticles that can be produced within the ferritin cage is remarkable and it is clear from the literature that careful experimental design and handling of the protein is key to the success of the deposition of ions within ferritin nanocages. Ferritin encapsulated metal nanoparticles can be liberated by removal of the ferritin cage by chemical or thermal means to leave highly homogeneous metal nanoparticles with uses ranging from the production of ordered semi-conductor arrays [10], quantum dots [41], and as anti-bacterial nanoparticles [42]. The use of mineralised ferritin in nanodevice fabrication and medical applications is discussed in more detail below.

The ability of ferritin to sequester phosphate within its core along with iron is well documented [43,44] and this can occur either simultaneously with iron sequestration, or after initial core formation [28]. This phenomenon has been applied to the removal of phosphate from water [28], where the *P. furiosus* ferritin has been developed as an industrial-scale solution to prevent water pollution and biofouling due to the phosphate content of seawater and industrial waste streams (www.biaqua.com). Current phosphate removal systems rely on the precipitation of phosphate using calcium and aluminium sulphates, or biological means where bacteria are used to sequester and adsorb calcium [45]. These chemical and biological methods have high maintenance costs, whereas the ferritin-based solution has a high capacity for phosphate binding, is highly stable because it is isolated from a thermophilic microorganism, and can be readily recycled [28]. The range of potential uses for this system is huge, but it remains to be seen whether it is widely adopted as an economical solution to phosphate removal and water treatment.

### Ferritin nanodevices

Both Ferritin and DPS have been used as scaffolds for the fabrication of inorganic nanodevices, such as quantum dots and nano-wires [3,10,12]. In these applications, ferritin cages loaded with mineral cores are deposited on silicon substrates that can be silanized and functionalised with small molecules, or peptides. The protein cage can be retained, or ablated with heat to leave the core in place. Patterned semi-conductor cores deposited on silicon wafers in this way have been used as memory gates [46,47]. Isolated metal cores have also been used as catalysts to seed the growth of carbon nano-tubes and nanowires [48]. These examples of the use of ferritins in the production of nanodevices are all at the proof of concept stage for technology development. It remains to be seen whether it will be possible to scale-up their production and implement ferritins in industrial applications at significant scale to have economic advantages over conventional semi-conductor fabrication methods.

### Ferritin based contrast agents for medical imaging

The plasticity of the *in vitro* mineralisation of ferritin makes it an ideal tool for cellular imaging, as labelled heavy atoms and heavy atom complexes can be readily sequestered within its core. Furthermore, the ability to modify ferritin through protein engineering and chemical means has enabled their use as contrast agents in basic scientific investigations of cellular ultrastructure and for medical imaging. Iron-loaded ferritin has also been used as a contrast agent in both electron microscopy [49] and MRI [50,51]. We will discuss applications of ferritin in these imaging modalities below.

Electron cryotomography (Cryo-ET) has become a valuable tool for studying the ultrastructure of cells and tissues as it allows them to be imaged at nanometre resolution in a near-native frozen-hydrated state. The low contrast of images produced by this method means that the identification of structures of interest is often challenging, therefore ferritin has been proposed as an electron-dense label for cryotomography. Ferritin fulfils the key requirement of a good cellular label as it has a significantly higher contrast than the cellular background due to the electron scattering properties of the iron core, and the ferritin cage is well ordered and homogeneous. Furthermore, surface modification of ferritin with fluorescent reporters, enables correlative fluorescence microscopy/Cryo-ET [49]. Using a multi-valent system like ferritin may produce sub-cellular artefacts when studying protein-protein interactions, especially at the cell surface, therefore care must be taken to control for the possibility of introducing this type of artefact. Because of its distinctive and highly homogeneous structure apo-ferritin has also been used as a standard for estimating the magnification of micrographs in Cryo-electron microscopy [52].

Magnetic Resonance Imaging (MRI) is a powerful diagnostic tool in both clinical and research settings, and is non-invasive and highly sensitive, with a resolution of up to 50 μm [53]. The most common MRI modality relies on the detection of ^1^H, which is abundant in tissues and gives a high quality signal that is positively influenced by the chemical environment of the atom. Exogeneous contrast agents are often used in conjunction with ^1^H to give additional information and to label specific tissues and track metabolic processes. Iron oxides and hydroxides, as found in the ferritin core, are efficient contrast agents due to their superparamagnetic properties and the dark contrast they give in MRI images [50].

The use of iron containing ferritin as an MRI contrast agent has been explored using lentiviral and adenovirus vectors encoding ferritin to transfect nerve cells in mice [51]. The transfected vectors produce ferritin within the targeted cells, which then accumulate excess iron because of the additional ferritin within them. This initial study highlights the importance of considering the delivery vehicle for ferritin and nanoparticles, as the results showed that the viral vectors themselves give enhanced MRI contrast due to the immune response against them at injection sites. Furthermore, due to the small size of ferritin, the authors note limited contrast against background signal *in vivo*.

Alternative strategies to the viral-vector induced expression of ferritin have been proposed that use recombinant ferritin tagged with targeting peptides and loaded with elements that give higher MRI contrast than iron, such as gadolinium [14]. Endothelial tumour cells were visualised by this strategy using gadolinium-loaded ferritin tagged with a peptide epitope specific for the neural cell adhesion molecule that is expressed on the tumour cells, but not healthy endothelial cells [54]. This approach gave a statistically significant enhancement to the MRI contrast localised specifically to the tumour cells. These studies highlight the promise of the use of ferritin as an MRI contrast agent if problems associated with the limited contrast available from native ferritin can be addressed. The production of labelled ferritin nanocages through the application of synthetic biology techniques for protein engineering will broaden the potential applications of this versatile scaffold.

### Drug delivery via the ferritin cavity

Further to the use of ferritin as an MRI contrast agent, other clinical applications for ferritin family proteins arise from their nature as highly stable compartments that are biocompatible, amenable to disassembly, reconstitution and surface modification. These properties have been used to develop ferritin as a drug delivery platform [15]. At physiological pH ferritin exists as a stable 24-mer, whilst in highly acidic or basic solutions it disassembles, and when returned to a neutral solution the complex spontaneously reassembles. This phenomenon can be used to trap molecules in solution within its cavity by dis/assembling ferritin in the presence of drug solutions. This property has been used to load the cavity with metal containing drugs, such as the cancer drug cisplatin, and the iron chelator desferrioxamine B [55,56], these drugs are readily encapsulated by ferritin due its natural tendency to bind to metals.

The incorporation of non-metal-containing drugs within ferritin is challenging due to the limited interactions between them and the ferritin shell, and the diffusion of these molecules through the surface pores. Strategies to overcome these problems have focused on complexing drugs with transition metals, such as Cu(II), prior to their internalisation [4], or the addition of charged accessory molecules such as poly-l-aspartic acid to optimise loading of ferritin with drugs [57]. By combining the loading of ferritin with drugs and surface modification with peptide epitopes and labels, ferritin can be specifically targeted to particular cell types and tumours for efficient delivery of therapeutic agents. There are limitations to the nature of the peptide labels and drugs that can be added to ferritin that limit the range of targeting molecules that can be used, but other protein-based nanocages have been demonstrated as alternative drug-delivery platforms [58]. The number of chemical processes involved in producing such delivery vectors may make such systems economically unviable, but where highly specific systems for the targeting of tumours are needed, ferritin and other protein nanocages show a great deal of promise that will hopefully transfer to a clinical setting.

### Ferritin nanoparticle vaccines

The role of nanotechnology in vaccine development is well established [59], as is the use of viruses for the display of heterologous proteins [2,60]. The highly symmetric and self-assembling ferritin nanocage presents an attractive target for vaccine development. In an elegant demonstration of the use of ferritin for vaccine development, Kanekiyo and colleagues fused the influenza virus haemagglutinin (**HA**) to the surface of *Helicobacter pylori* ferritin [16]. Fusions of entire protein domains to the surface of ferritin are complicated by the requirement to match the subunit organisation of the protein to be displayed on its surface with the inherent symmetry of the ferritin cage itself. HA forms trimers with 30 Å between the central axes of each subunit, this matches the distance between the N-terminal residues of the *H. pylori* ferritin structure, which are found at the 3-fold symmetry axis of the ferritin cage. Immunisation of mice with the HA-Ftn nanoparticle vaccine elicited the production of broadly neutralising antibodies against different HA variants and showed enhanced potency compared to a commercially available influenza vaccine, with no autoimmune reaction due to the use of the *H. pylori* ferritin whose sequence is highly divergent from human ferritin and which incorporates limited quantities of iron when produced heterologously [61]. This study highlights the potential benefits of producing and implementing synthetic nanoparticle vaccines, but a degree of caution is required to avoid autoimmune reactions caused by the vector itself. The use of such vaccines would abrogate the need to generate live viruses in cell-culture, and when coupled with synthetic biology methods would allow multi-component vaccines with broad activities to be produced entirely recombinantly [62].

## Future prospects

Medical imaging shows the greatest immediate prospects for widespread adoption of the bionanotechnology applications of ferritin presented in this review. The use of ferritin derivatives has potential to transform the diagnosis and treatment of tumours *in situ*. A key limitation of this application is the poor MRI contrast achieved by the native iron containing ferritin [51]; recent research on a family of protein nanocompartments known as encapsulins has identified a new class of ferritin-like proteins that are selectively enclosed within the encapsulin shell, which is much larger than the classical ferritin and may present a solution to this contrast problem [63,64]. McHugh and colleagues propose that the encapsulins function as an iron mega-store as their internal cavities can accommodate up to 10 times more mineralised iron than ferritin [64]. It is in fact possible to sequester ferritin within an encapsulin shell [65] and while this has been demonstrated in a recombinant system it is not clear whether this is a physiologically relevant arrangement. The compartmentalisation of EncFtn (encapsulated Ftn) within the encapsulin shell is mediated by short C-terminal localisation sequences, which can be appended to heterologous proteins to direct them to the encapsulin interior [66]. This feature of encapsulins makes them highly attractive platforms for exploitation as cell factories, or to protect unstable protein cargoes.

In this review we have shown a range of technologies that exploit the features of ferritin and ferritin family proteins and have given examples drawn from current research that are exploring ways to make use the unique properties of ferritins. The use of thermostable ferritin from *P. furiosus* in water treatment to remove phosphate is the only process-scale use of ferritin nanocompartments to date; while the other examples presented are still at the stage of laboratory-scale technology demonstrations [28]. The question of whether ferritin-based semi-conductor and medical systems will be used outside of the research laboratory rests on economic factors relating to the cost of implementing these technologies over the current state-of-the art in these fields. The recent discovery of encapsulated ferritins and their protein containers has identified an exciting new platform for use in bionanotechnology; the use of synthetic biology tools will enable their rapid implementation in materials science, biotechnology, and medical applications.

## Figures and Tables

**Table 1:** Applications and sources of ferritin used in bionanotechnology

**Application**	**Core composition**	**Source**	**Reference**
**MRI contrast agent**	Iron oxyhydroxide	Transfected human H-chain ferritin	[38]
	Iron oxyhydroxide	Adenovirus/lentivirus vector	[47]
**Phosphate removal from water**	Ferric phosphate	*Pyrococcus furiosus* ferritin	[28]
**Quantum label**	ZnSe	Horse spleen apoferritin	[59]
**Semi-conductor template**	CdS/ZnSe	Apoferritin	[60]
**Antibacterial silver nanoparticles**	Ag	*Pyrococcus furiosus* ferritin	[37]
		Engineered human H-chain ferritin	[35]
**Gold nanoparticles**	Au	Engineered human H-chain ferritin	[35]
**Drug delivery**	Doxorubicin–Cu(II) complex	Surface-modified human H-chain ferritin	[15]
**Information storage**	CoPt	Horse spleen apoferritin	[67]
		*Pyrococcus furiosus* ferritin	[33]
**Magnetic nanoparticles**	Co_3_O_4_	Horse spleen apoferritin	[68]
**Vaccine development**	None	Helicobacter pylori non-heam ferritin	[61]
**Chemical catalyst**	Pt	Apoferritin from Sigma	[69]
	Pd	Recombinant L-chain apoferritin from horse liver	[36]
	Cu	Horse spleen apoferritin	[70]
	Au	Horse spleen apoferritin	[31]
	TiO_2_	Mammalian apoferritin	[38]
